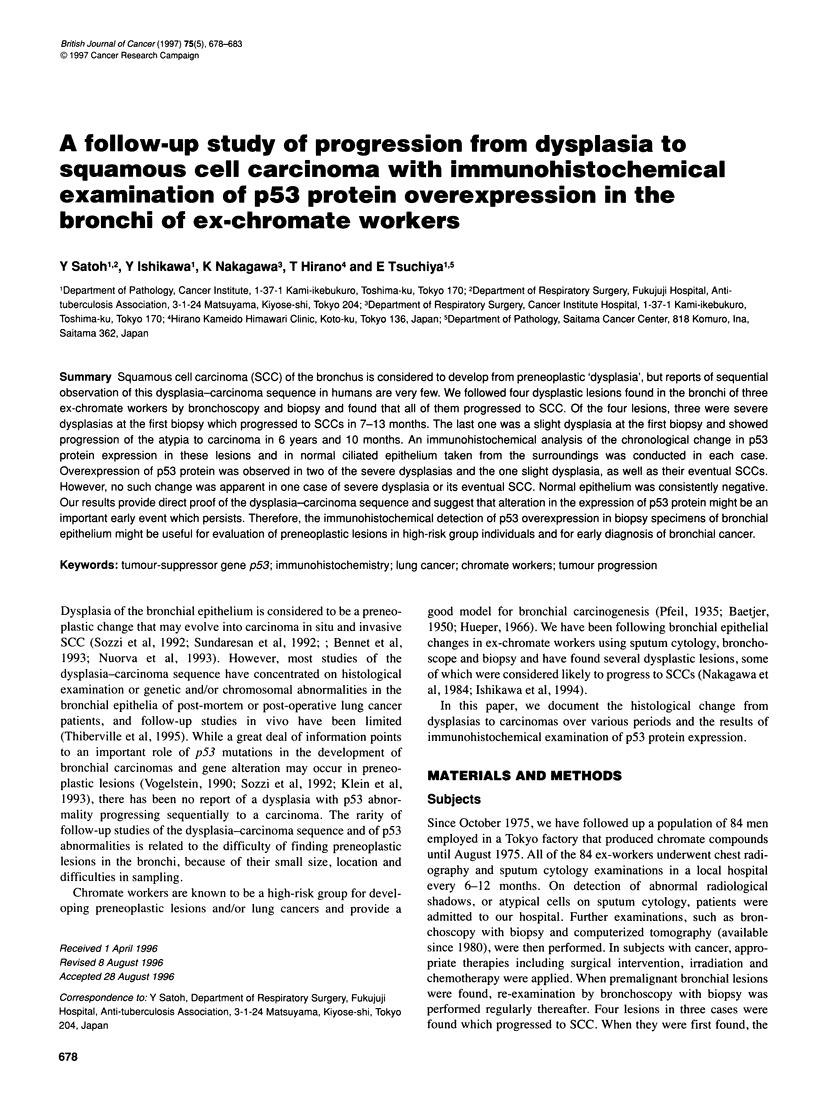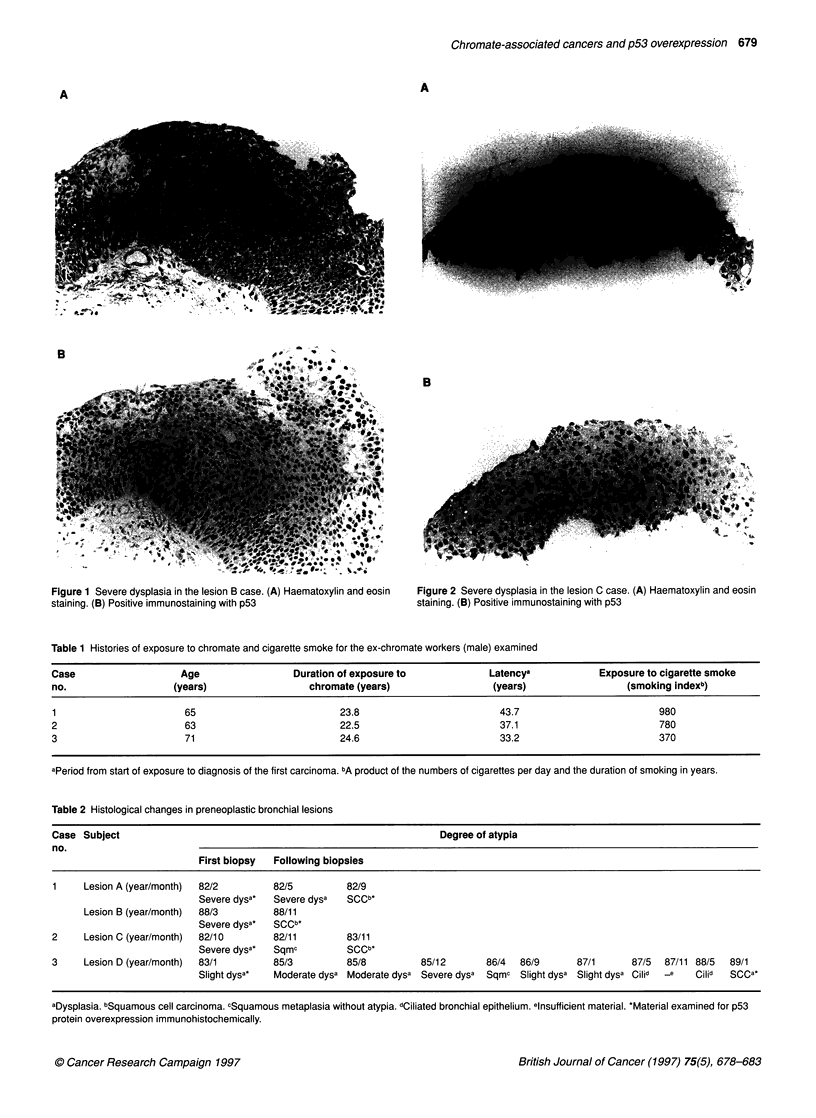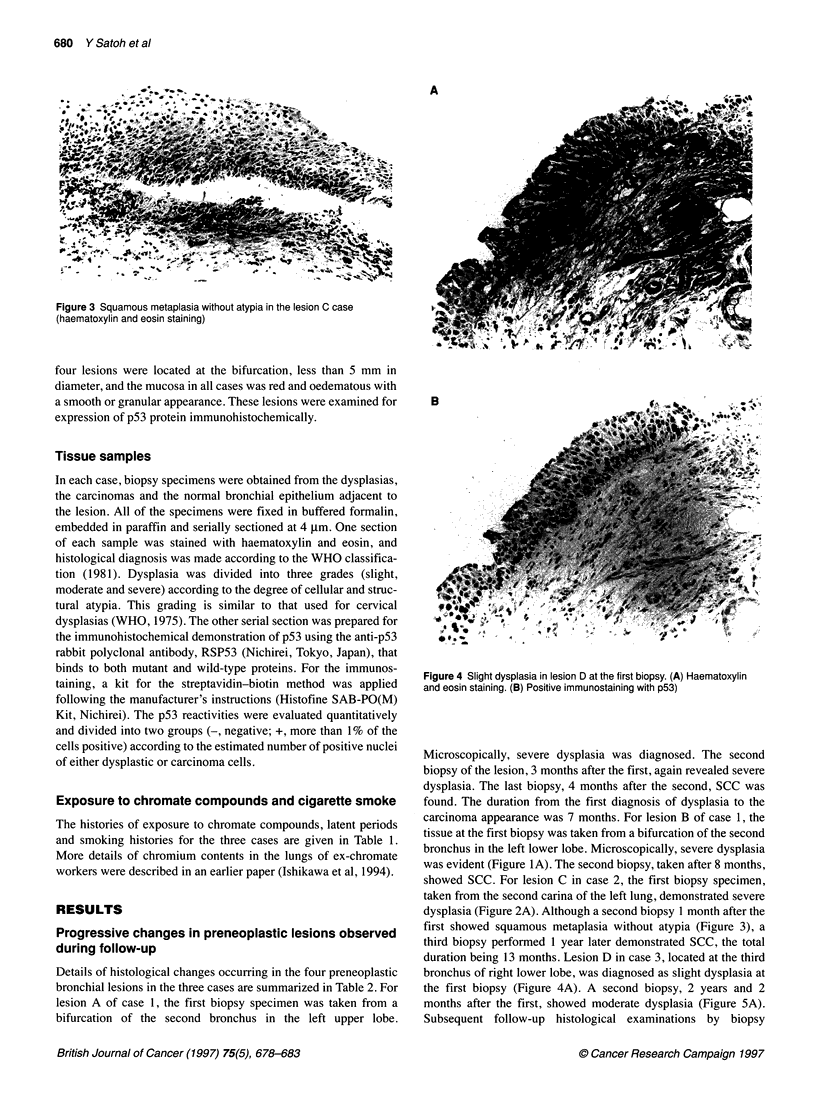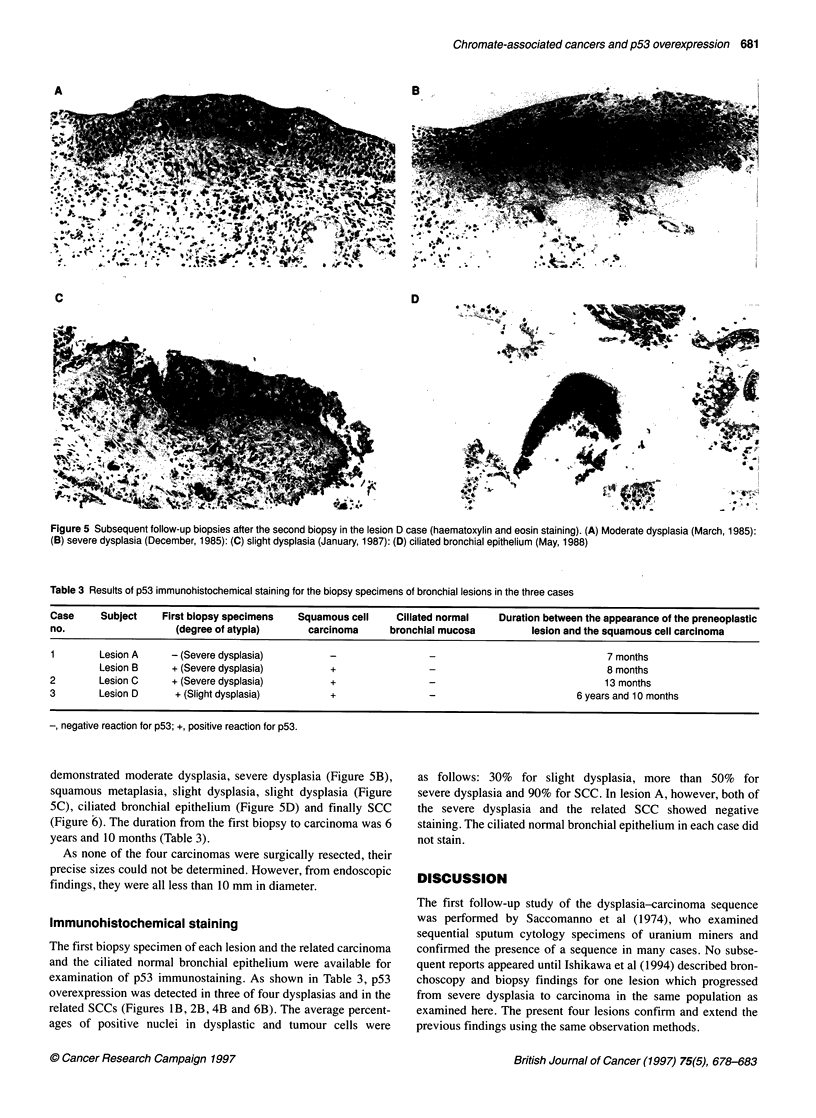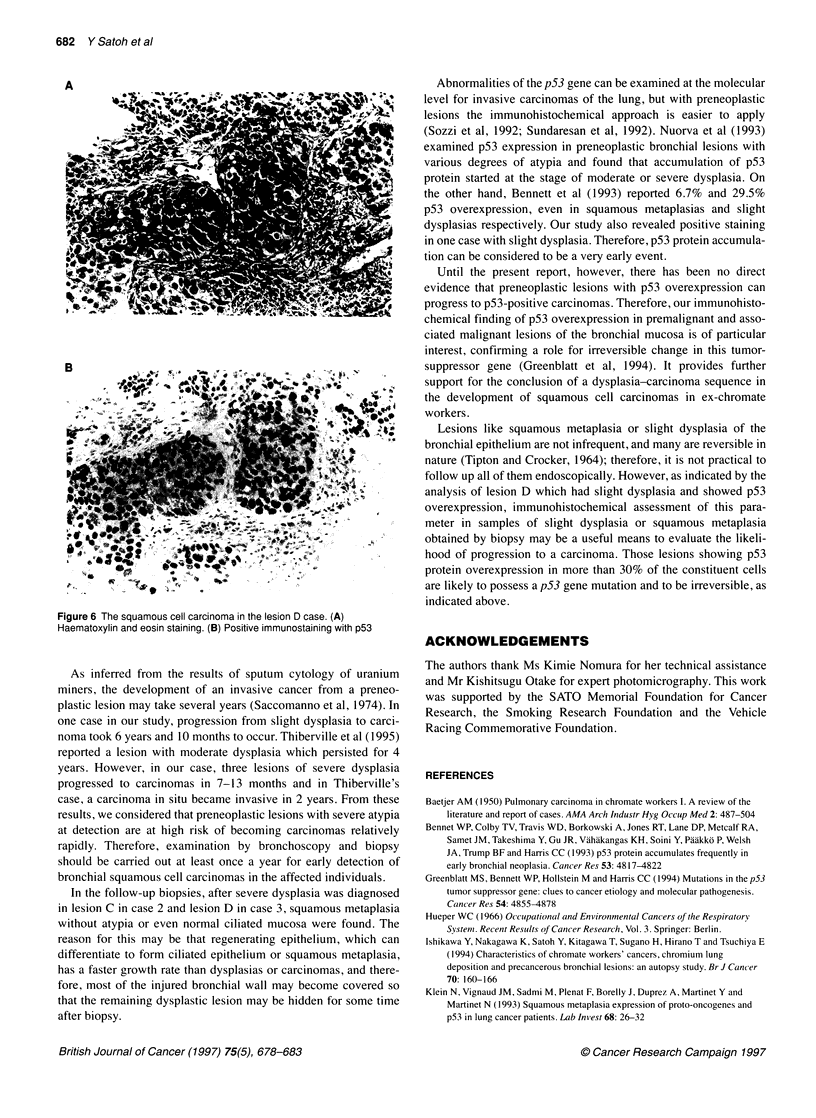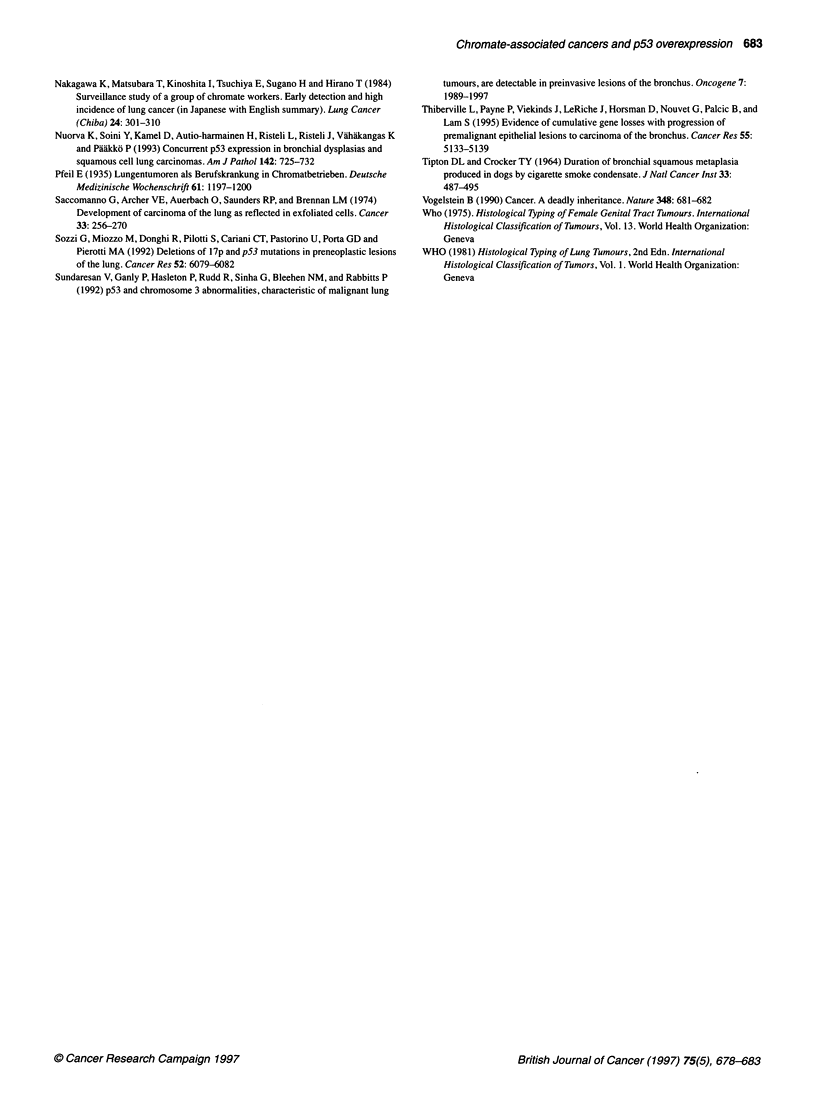# A follow-up study of progression from dysplasia to squamous cell carcinoma with immunohistochemical examination of p53 protein overexpression in the bronchi of ex-chromate workers.

**DOI:** 10.1038/bjc.1997.121

**Published:** 1997

**Authors:** Y. Satoh, Y. Ishikawa, K. Nakagawa, T. Hirano, E. Tsuchiya

**Affiliations:** Department of Pathology, Cancer Institute, Toshima-ku, Tokyo, Japan.

## Abstract

**Images:**


British Journal of Cancer (1997) 75(5), 678–683
© 1997 Cancer Research Campaign

### A follow-up study of progression from dysplasia to squamous cell carcinoma with immunohistochemical examination of p53 protein overexpression in the bronchi of ex-chromate workers

**Y Satoh[1,2], Y Ishikawa[1], K Nakagawa[3], T Hirano[4] and E Tsuchiya[1,5]**

[1]Department of Pathology, Cancer Institute, 1-37-1 Kami-ikebukuro, Toshima-ku, Tokyo 170; [2]Department of Respiratory Surgery, Fukujuji Hospital, Anti-tuberculosis Association, 3-1-24 Matsuyama, Kiyose-shi, Tokyo 204; [3]Department of Respiratory Surgery, Cancer Institute Hospital, 1-37-1 Kami-ikebukuro, Toshima-ku, Tokyo 170; [4]Hirano Kameido Himawari Clinic, Koto-ku, Tokyo 136, Japan; [5]Department of Pathology, Saitama Cancer Center, 818 Komuro, Ina, Saitama 362, Japan

**Summary** Squamous cell carcinoma (SCC) of the bronchus is considered to develop from preneoplastic 'dysplasia', but reports of sequential observation of this dysplasia–carcinoma sequence in humans are very few. We followed four dysplastic lesions found in the bronchi of three ex-chromate workers by bronchoscopy and biopsy and found that all of them progressed to SCC. Of the four lesions, three were severe dysplasias at the first biopsy which progressed to SCCs in 7–13 months. The last one was a slight dysplasia at the first biopsy and showed progression of the atypia to carcinoma in 6 years and 10 months. An immunohistochemical analysis of the chronological change in p53 protein expression in these lesions and in normal ciliated epithelium taken from the surroundings was conducted in each case. Overexpression of p53 protein was observed in two of the severe dysplasias and the one slight dysplasia, as well as their eventual SCCs. However, no such change was apparent in one case of severe dysplasia or its eventual SCC. Normal epithelium was consistently negative. Our results provide direct proof of the dysplasia–carcinoma sequence and suggest that alteration in the expression of p53 protein might be an important early event which persists. Therefore, the immunohistochemical detection of p53 overexpression in biopsy specimens of bronchial epithelium might be useful for evaluation of preneoplastic lesions in high-risk group individuals and for early diagnosis of bronchial cancer.

**Keywords:** tumour-suppressor gene *p53*; immunohistochemistry; lung cancer; chromate workers; tumour progression

Dysplasia of the bronchial epithelium is considered to be a preneoplastic change that may evolve into carcinoma in situ and invasive SCC (Sozzi et al, 1992; Sundaresan et al, 1992; ; Bennet et al, 1993; Nuorva et al, 1993). However, most studies of the dysplasia–carcinoma sequence have concentrated on histological examination or genetic and/or chromosomal abnormalities in the bronchial epithelia of post-mortem or post-operative lung cancer patients, and follow-up studies in vivo have been limited (Thiberville et al, 1995). While a great deal of information points to an important role of *p53* mutations in the development of bronchial carcinomas and gene alteration may occur in preneoplastic lesions (Vogelstein, 1990; Sozzi et al, 1992; Klein et al, 1993), there has been no report of a dysplasia with p53 abnormality progressing sequentially to a carcinoma. The rarity of follow-up studies of the dysplasia–carcinoma sequence and of p53 abnormalities is related to the difficulty of finding preneoplastic lesions in the bronchi, because of their small size, location and difficulties in sampling.

Chromate workers are known to be a high-risk group for developing preneoplastic lesions and/or lung cancers and provide a good model for bronchial carcinogenesis (Pfeil, 1935; Baetjer, 1950; Hueper, 1966). We have been following bronchial epithelial changes in ex-chromate workers using sputum cytology, bronchoscope and biopsy and have found several dysplastic lesions, some of which were considered likely to progress to SCCs (Nakagawa et al, 1984; Ishikawa et al, 1994).

In this paper, we document the histological change from dysplasias to carcinomas over various periods and the results of immunohistochemical examination of p53 protein expression.

*Received 1 April 1996*
*Revised 8 August 1996*
*Accepted 28 August 1996*

*Correspondence to:* Y Satoh, Department of Respiratory Surgery, Fukujuji Hospital, Anti-tuberculosis Association, 3-1-24 Matsuyama, Kiyose-shi, Tokyo 204, Japan

### MATERIALS AND METHODS

### Subjects

Since October 1975, we have followed up a population of 84 men employed in a Tokyo factory that produced chromate compounds until August 1975. All of the 84 ex-workers underwent chest radiography and sputum cytology examinations in a local hospital every 6–12 months. On detection of abnormal radiological shadows, or atypical cells on sputum cytology, patients were admitted to our hospital. Further examinations, such as bronchoscopy with biopsy and computerized tomography (available since 1980), were then performed. In subjects with cancer, appropriate therapies including surgical intervention, irradiation and chemotherapy were applied. When premalignant bronchial lesions were found, re-examination by bronchoscopy with biopsy was performed regularly thereafter. Four lesions in three cases were found which progressed to SCC. When they were first found, the

Figure 1 Severe dysplasia in the lesion B case. (**A**) Haematoxylin and eosin staining. (**B**) Positive immunostaining with p53

Figure 2 Severe dysplasia in the lesion C case. (**A**) Haematoxylin and eosin staining. (**B**) Positive immunostaining with p53

**Table 1** Histories of exposure to chromate and cigarette smoke for the ex-chromate workers (male) examined

| Case no. | Age (years) | Duration of exposure to chromate (years) | Latency[a] (years) | Exposure to cigarette smoke (smoking index[b]) |
|---|---|---|---|---|
| 1 | 65 | 23.8 | 43.7 | 980 |
| 2 | 63 | 22.5 | 37.1 | 780 |
| 3 | 71 | 24.6 | 33.2 | 370 |

[a]Period from start of exposure to diagnosis of the first carcinoma. [b]A product of the numbers of cigarettes per day and the duration of smoking in years.

**Table 2** Histological changes in preneoplastic bronchial lesions

| Case no. | Subject | Degree of atypia | | | | | | | | | | |
|---|---|---|---|---|---|---|---|---|---|---|---|---|
| | | First biopsy | Following biopsies | | | | | | | | | |
| 1 | Lesion A (year/month) | 82/2 | 82/5 | 82/9 | | | | | | | | |
| | | Severe dys[a]* | Severe dys[a] | SCC[b]* | | | | | | | | |
| | Lesion B (year/month) | 88/3 | 88/11 | | | | | | | | | |
| | | Severe dys[a]* | SCC[b]* | | | | | | | | | |
| 2 | Lesion C (year/month) | 82/10 | 82/11 | 83/11 | | | | | | | | |
| | | Severe dys[a]* | Sqm[c] | SCC[b]* | | | | | | | | |
| 3 | Lesion D (year/month) | 83/1 | 85/3 | 85/8 | 85/12 | 86/4 | 86/9 | 87/1 | 87/5 | 87/11 | 88/5 | 89/1 |
| | | Slight dys[a]* | Moderate dys[a] | Moderate dys[a] | Severe dys[a] | Sqm[c] | Slight dys[a] | Slight dys[a] | Cili[d] | –[e] | Cili[d] | SCC[a]* |

[a]Dysplasia. [b]Squamous cell carcinoma. [c]Squamous metaplasia without atypia. [d]Ciliated bronchial epithelium. [e]Insufficient material. *Material examined for p53 protein overexpression immunohistochemically.

© Cancer Research Campaign 1997

Figure 3 Squamous metaplasia without atypia in the lesion C case (haematoxylin and eosin staining)

four lesions were located at the bifurcation, less than 5 mm in diameter, and the mucosa in all cases was red and oedematous with a smooth or granular appearance. These lesions were examined for expression of p53 protein immunohistochemically.

### Tissue samples

In each case, biopsy specimens were obtained from the dysplasias, the carcinomas and the normal bronchial epithelium adjacent to the lesion. All of the specimens were fixed in buffered formalin, embedded in paraffin and serially sectioned at 4 µm. One section of each sample was stained with haematoxylin and eosin, and histological diagnosis was made according to the WHO classification (1981). Dysplasia was divided into three grades (slight, moderate and severe) according to the degree of cellular and structural atypia. This grading is similar to that used for cervical dysplasias (WHO, 1975). The other serial section was prepared for the immunohistochemical demonstration of p53 using the anti-p53 rabbit polyclonal antibody, RSP53 (Nichirei, Tokyo, Japan), that binds to both mutant and wild-type proteins. For the immunostaining, a kit for the streptavidin–biotin method was applied following the manufacturer's instructions (Histofine SAB-PO(M) Kit, Nichirei). The p53 reactivities were evaluated quantitatively and divided into two groups (–, negative; +, more than 1% of the cells positive) according to the estimated number of positive nuclei of either dysplastic or carcinoma cells.

### Exposure to chromate compounds and cigarette smoke

The histories of exposure to chromate compounds, latent periods and smoking histories for the three cases are given in Table 1. More details of chromium contents in the lungs of ex-chromate workers were described in an earlier paper (Ishikawa et al, 1994).

### RESULTS

### Progressive changes in preneoplastic lesions observed during follow-up

Details of histological changes occurring in the four preneoplastic bronchial lesions in the three cases are summarized in Table 2. For lesion A of case 1, the first biopsy specimen was taken from a bifurcation of the second bronchus in the left upper lobe. Microscopically, severe dysplasia was diagnosed. The second biopsy of the lesion, 3 months after the first, again revealed severe dysplasia. The last biopsy, 4 months after the second, SCC was found. The duration from the first diagnosis of dysplasia to the carcinoma appearance was 7 months. For lesion B of case 1, the tissue at the first biopsy was taken from a bifurcation of the second bronchus in the left lower lobe. Microscopically, severe dysplasia was evident (Figure 1A). The second biopsy, taken after 8 months, showed SCC. For lesion C in case 2, the first biopsy specimen, taken from the second carina of the left lung, demonstrated severe dysplasia (Figure 2A). Although a second biopsy 1 month after the first showed squamous metaplasia without atypia (Figure 3), a third biopsy performed 1 year later demonstrated SCC, the total duration being 13 months. Lesion D in case 3, located at the third bronchus of right lower lobe, was diagnosed as slight dysplasia at the first biopsy (Figure 4A). A second biopsy, 2 years and 2 months after the first, showed moderate dysplasia (Figure 5A). Subsequent follow-up histological examinations by biopsy

A

B

Figure 4 Slight dysplasia in lesion D at the first biopsy. (**A**) Haematoxylin and eosin staining. (**B**) Positive immunostaining with p53)

© Cancer Research Campaign 1997

**Figure 5** Subsequent follow-up biopsies after the second biopsy in the lesion D case (haematoxylin and eosin staining). (**A**) Moderate dysplasia (March, 1985): (**B**) severe dysplasia (December, 1985): (**C**) slight dysplasia (January, 1987): (**D**) ciliated bronchial epithelium (May, 1988)

**Table 3** Results of p53 immunohistochemical staining for the biopsy specimens of bronchial lesions in the three cases

| Case no. | Subject | First biopsy specimens (degree of atypia) | Squamous cell carcinoma | Ciliated normal bronchial mucosa | Duration between the appearance of the preneoplastic lesion and the squamous cell carcinoma |
|---|---|---|---|---|---|
| 1 | Lesion A | – (Severe dysplasia) | – | – | 7 months |
| | Lesion B | + (Severe dysplasia) | + | – | 8 months |
| 2 | Lesion C | + (Severe dysplasia) | + | – | 13 months |
| 3 | Lesion D | + (Slight dysplasia) | + | – | 6 years and 10 months |

–, negative reaction for p53; +, positive reaction for p53.

demonstrated moderate dysplasia, severe dysplasia (Figure 5B), squamous metaplasia, slight dysplasia, slight dysplasia (Figure 5C), ciliated bronchial epithelium (Figure 5D) and finally SCC (Figure 6). The duration from the first biopsy to carcinoma was 6 years and 10 months (Table 3).

As none of the four carcinomas were surgically resected, their precise sizes could not be determined. However, from endoscopic findings, they were all less than 10 mm in diameter.

### Immunohistochemical staining

The first biopsy specimen of each lesion and the related carcinoma and the ciliated normal bronchial epithelium were available for examination of p53 immunostaining. As shown in Table 3, p53 overexpression was detected in three of four dysplasias and in the related SCCs (Figures 1B, 2B, 4B and 6B). The average percentages of positive nuclei in dysplastic and tumour cells were as follows: 30% for slight dysplasia, more than 50% for severe dysplasia and 90% for SCC. In lesion A, however, both of the severe dysplasia and the related SCC showed negative staining. The ciliated normal bronchial epithelium in each case did not stain.

### DISCUSSION

The first follow-up study of the dysplasia–carcinoma sequence was performed by Saccomanno et al (1974), who examined sequential sputum cytology specimens of uranium miners and confirmed the presence of a sequence in many cases. No subsequent reports appeared until Ishikawa et al (1994) described bronchoscopy and biopsy findings for one lesion which progressed from severe dysplasia to carcinoma in the same population as examined here. The present four lesions confirm and extend the previous findings using the same observation methods.

© Cancer Research Campaign 1997

Abnormalities of the *p53* gene can be examined at the molecular level for invasive carcinomas of the lung, but with preneoplastic lesions the immunohistochemical approach is easier to apply (Sozzi et al, 1992; Sundaresan et al, 1992). Nuorva et al (1993) examined p53 expression in preneoplastic bronchial lesions with various degrees of atypia and found that accumulation of p53 protein started at the stage of moderate or severe dysplasia. On the other hand, Bennett et al (1993) reported 6.7% and 29.5% p53 overexpression, even in squamous metaplasias and slight dysplasias respectively. Our study also revealed positive staining in one case with slight dysplasia. Therefore, p53 protein accumulation can be considered to be a very early event.

Until the present report, however, there has been no direct evidence that preneoplastic lesions with p53 overexpression can progress to p53-positive carcinomas. Therefore, our immunohistochemical finding of p53 overexpression in premalignant and associated malignant lesions of the bronchial mucosa is of particular interest, confirming a role for irreversible change in this tumor-suppressor gene (Greenblatt et al, 1994). It provides further support for the conclusion of a dysplasia–carcinoma sequence in the development of squamous cell carcinomas in ex-chromate workers.

Lesions like squamous metaplasia or slight dysplasia of the bronchial epithelium are not infrequent, and many are reversible in nature (Tipton and Crocker, 1964); therefore, it is not practical to follow up all of them endoscopically. However, as indicated by the analysis of lesion D which had slight dysplasia and showed p53 overexpression, immunohistochemical assessment of this parameter in samples of slight dysplasia or squamous metaplasia obtained by biopsy may be a useful means to evaluate the likelihood of progression to a carcinoma. Those lesions showing p53 protein overexpression in more than 30% of the constituent cells are likely to possess a *p53* gene mutation and to be irreversible, as indicated above.

### ACKNOWLEDGEMENTS

The authors thank Ms Kimie Nomura for her technical assistance and Mr Kishitsugu Otake for expert photomicrography. This work was supported by the SATO Memorial Foundation for Cancer Research, the Smoking Research Foundation and the Vehicle Racing Commemorative Foundation.

### REFERENCES

Baetjer AM (1950) Pulmonary carcinoma in chromate workers I. A review of the literature and report of cases. *AMA Arch Industr Hyg Occup Med* **2**: 487–504

Bennet WP, Colby TV, Travis WD, Borkowski A, Jones RT, Lane DP, Metcalf RA, Samet JM, Takeshima Y, Gu JR, Vähäkangas KH, Soini Y, Pääkkö P, Welsh JA, Trump BF and Harris CC (1993) p53 protein accumulates frequently in early bronchial neoplasia. *Cancer Res* **53**: 4817–4822

Greenblatt MS, Bennett WP, Hollstein M and Harris CC (1994) Mutations in the *p53* tumor suppressor gene: clues to cancer etiology and molecular pathogenesis. *Cancer Res* **54**: 4855–4878

Hueper WC (1966) *Occupational and Environmental Cancers of the Respiratory System. Recent Results of Cancer Research*, Vol. 3. Springer: Berlin.

Ishikawa Y, Nakagawa K, Satoh Y, Kitagawa T, Sugano H, Hirano T and Tsuchiya E (1994) Characteristics of chromate workers' cancers, chromium lung deposition and precancerous bronchial lesions: an autopsy study. *Br J Cancer* **70**: 160–166

Klein N, Vignaud JM, Sadmi M, Plenat F, Borelly J, Duprez A, Martinet Y and Martinet N (1993) Squamous metaplasia expression of proto-oncogenes and p53 in lung cancer patients. *Lab Invest* **68**: 26–32

© Cancer Research Campaign 1997

Nakagawa K, Matsubara T, Kinoshita I, Tsuchiya E, Sugano H and Hirano T (1984) Surveillance study of a group of chromate workers. Early detection and high incidence of lung cancer (in Japanese with English summary). *Lung Cancer (Chiba)* **24**: 301–310

Nuorva K, Soini Y, Kamel D, Autio-harmainen H, Risteli L, Risteli J, Vähäkangas K and Pääkkö P (1993) Concurrent p53 expression in bronchial dysplasias and squamous cell lung carcinomas. *Am J Pathol* **142**: 725–732

Pfeil E (1935) Lungentumoren als Berufskrankung in Chromatbetrieben. *Deutsche Medizinische Wochenschrift* **61**: 1197–1200

Saccomanno G, Archer VE, Auerbach O, Saunders RP, and Brennan LM (1974) Development of carcinoma of the lung as reflected in exfoliated cells. *Cancer* **33**: 256–270

Sozzi G, Miozzo M, Donghi R, Pilotti S, Cariani CT, Pastorino U, Porta GD and Pierotti MA (1992) Deletions of 17p and *p53* mutations in preneoplastic lesions of the lung. *Cancer Res* **52**: 6079–6082

Sundaresan V, Ganly P, Hasleton P, Rudd R, Sinha G, Bleehen NM, and Rabbitts P (1992) p53 and chromosome 3 abnormalities, characteristic of malignant lung tumours, are detectable in preinvasive lesions of the bronchus. *Oncogene* **7**: 1989–1997

Thiberville L, Payne P, Viekinds J, LeRiche J, Horsman D, Nouvet G, Palcic B, and Lam S (1995) Evidence of cumulative gene losses with progression of premalignant epithelial lesions to carcinoma of the bronchus. *Cancer Res* **55**: 5133–5139

Tipton DL and Crocker TY (1964) Duration of bronchial squamous metaplasia produced in dogs by cigarette smoke condensate. *J Natl Cancer Inst* **33**: 487–495

Vogelstein B (1990) Cancer. A deadly inheritance. *Nature* **348**: 681–682

Who (1975). *Histological Typing of Female Genital Tract Tumours. International Histological Classification of Tumours*, Vol. 13. World Health Organization: Geneva

WHO (1981) *Histological Typing of Lung Tumours*, 2nd Edn. *International Histological Classification of Tumors*, Vol. 1. World Health Organization: Geneva

© Cancer Research Campaign 1997